# Prevalence and change of central obesity among US Asian adults: NHANES 2011–2014

**DOI:** 10.1186/s12889-017-4689-6

**Published:** 2017-08-25

**Authors:** Xuefeng Liu, Yang Chen, Nicole L. Boucher, Amy E. Rothberg

**Affiliations:** 1 Department of Systems, Population, and Leadership, University of Michigan, Ann Arbor, MI 48109 USA; 2 Frankel Cardiovascular Center, University of Michigan School of Medicine, Ann Arbor, MI 48109 USA; 3 Department of Health Behavior and Biological Sciences, University of Michigan, Ann Arbor, MI 48109 USA; 4 Department of Internal Medicine, Division of Metabolism, Endocrinology & Diabetes, University of Michigan, Ann Arbor, MI 48109 USA; 5 Department of Nutritional Sciences, School of Public Health, University of Michigan, Ann Arbor, MI 48109 USA; 6 Department of Systems, Population, and Leadership, University of Michigan School of Nursing, 400 N. Ingalls, Ann Arbor, MI 48109-5482 USA

**Keywords:** Central obesity, Prevalence, Asian adults

## Abstract

**Background:**

Central obesity is a major risk factor for cardiometabolic diseases. The prevalence of central obesity has not been reported fully among Asian adults in the United States (US).

**Methods:**

Cross-sectional data of 1288 Asian adults aged 20 years or over was selected from the US National Health and Nutrition Examination Survey with a stratified multi-stage sampling design. The prevalence of central obesity was calculated with 95% confidence intervals (CIs) and Chi-square tests were conducted to test the significance of the prevalence differences across characteristic groups.

**Results:**

The overall prevalence of central obesity among US Asian adults was 58.1% in 2011–2014. The prevalence of central obesity was higher in older adults (73.5%) than in young adults (45.4%) (*p* < 0.0001). Women had 13.4% higher prevalence than men (64.4% vs 51.0%, *p* < 0.0001). The prevalence increased over time (2011–2012 vs 2013–2014) in young adults (39.2% vs 51.5%), men (45.4% vs 56.6%), adults with college education or above (54.2% vs 61.7%) and non-poor adults (55.4% vs 62.4%). Compared with men, women had higher prevalence in each subgroup of age, education, poverty, and length of time (except for the subgroup of “born in the US”) (all *p* < 0.05) and in the subgroup of “married or living with partner” for marital status (*p* < 0.0001).

**Conclusion:**

Central obesity is prevalent in Asian adults, particularly in older adults and women. More efforts are needed to prevent and treat obesity in Asian adults as Asians are incurring the greatest increase in type 2 diabetes in parallel with the rising rate of central adiposity.

## Background

Body mass index (BMI) has been used widely to assess general obesity which is a major risk factor for cardiometabolic disease and overall deaths in the United States (US) [1]. Yet BMI has limitations in predicting obesity-related health risks, especially at lower level of BMI [2]. Waist circumference (WC) is a sensitive indicator of body fat distribution that is considered to characterize central obesity [3]. Many studies shows that central obesity correlates higher with hypertension, diabetes, dyslipidemia, metabolic syndrome, and coronary heart disease independent of BMI [4–6]. The US National Institutes of Health recommends that WC, in addition to weight and height, be measured in primary care practice to determine the risk of weight-related complications and guide decision-making for weight management intervention [7].

Asians from south Asia have high prevalence of diabetes mellitus, hypertension, and cardiovascular disease (CVD), despite low levels of BMI [8–10]. There has been a rapid growth of cardiometabolic risks and CVD in the US Asian population [10–12]. Central obesity may play an important role in these increased risks. As the Asian population is the fastest growing ethnic group in the US [13], Information on obesity with emphasis on central obesity would be useful for determining the risk with interventions targeted at reducing obesity-related complications and enhancing health-related quality of life in Asian adults.

Although the prevalence of central obesity has been reported in several populations [14–17], it has not been fully investigated in the US Asian population. Ford et al. have reported trends in the prevalence of central obesity among US adults and presented the general prevalence by sex in the Asian population [18]. However, there are two major concerns in their study. One is that they have defined the central obesity for Asians using the criteria for white and black adults which may not be appropriate for Asian adults. The other is that they have focused on central obesity in the general US population but not Asians (mostly non-Hispanic white, non-Hispanic black and Mexican American), and did not address the difference in the prevalence of central obesity over other characteristics except for sex among US Asian adults.

In this study, data on WC measurements and demographic characteristics were extracted from the National Health and Nutrition Examination Survey (NHANES) 2011–2014. The prevalence of central obesity and the difference in the prevalence across demographic and socioeconomic groups were examined among US Asian adults. The results will provide meaningful information on central obesity that can be used to compare the US Asian population with other populations (e.g. US white, black or Mexican American population) to pinpoint the potential role of fat distributions in racial/ethnic disparities in adverse health outcomes.

## Methods

The continuous NHANES, beginning in 1999, includes a series of two-year health and nutrition surveys conducted by the National Center for Health Statistics (NCHS) in the Centers for Disease Control and Prevention (CDC). Each survey used a stratified multistage-clustered sampling design to select the sample of participants, representative of the US civilian noninstitutionalized resident population. The survey consisted of interview questionnaires and health examinations. Interviews were performed in participants’ homes and elicited information pertaining to demographic, socioeconomic, dietary, and health-related status. Physical examinations and laboratory blood draws were conducted in the mobile examination centers to obtain medical and dental information and anthropometric measurements. All participants provided written informed consent and the data was approved by the NCHS Institutional/Ethics Review Board to ensure human subject protection and confidentiality [19].

### Study participants

All participants in the present study were non-Hispanic Asians from NHANES 2011–2014. This study focused on central obesity among US Asians and NCHS only began to collect data on Asians in 2011 [19]. Non-Hispanic Asians included all persons having origins in the Far East, Southeast Asia, or the Indian subcontinent (including, for example, China, Japan, Korea, Malaysia, the Philippine Islands, Vietnam, Thailand, Cambodia, India, Pakistan,). Our sample did not include Asians with multi-racial/ethnic background. In 2011–2014, primary sample design changes were implemented in NHANES to oversample non-Hispanic Asians and increase the reliability and precision of health estimates for this subgroup [19]. Participants who were less than 20 years were excluded from the study. Pregnant women and those who did not have a WC recorded were also excluded. The final study sample consisted of 1288 non-Hispanic Asian adults.

### WC measurements and central obesity

WC was measured by trained examiners following body measures examination protocol [20]. The measurement room was equipped with wall mirrors designed to facilitate accurate and efficient measurements. A health technologist and recorder worked as a team to assist the examiners. WC data were saved to the study database using the Integrated Survey Information System (ISIS) anthropometry computer application. Each eligible participant was measured without clothing and stood in the pose of crossing arms with hands on opposite shoulders. The examiner made a mark just above the uppermost lateral border of the right ilium of the participant, and then extended the measuring tape around the waist and positioned it in a horizontal plane at the level of the measurement mark. The measuring tape was placed parallel to the floor, and fit snug across but did not compress the skin. WC was taken to the nearest 0.1 cm at the end of the participant’s normal expiration. Central obesity was defined as WC ≥90 cm for men and ≥80 cm for women according to the guidelines of the International Diabetes Federation (IDF) for Asian populations [21].

### Demographic and socioeconomic characteristics

All the characteristics considered in this study were self-reported through interview questionnaires. Age and gender were demographic factors; education, poverty status, and marital status were socioeconomic factors. Length of stay in the US was used to reflect the acculturation including cultural, psychological, and lifestyle changes. We considered these characteristics to examine how the prevalence of central obesity differed across the groups defined by these characteristics. The selected participants were categorized into three groups: young adults (aged 20–39 years), middle-aged adults (aged 40–59 years), and old adults (aged 60 years or above). In general, 20–39 year olds are more similar phenotypically to each other than they are to those who are middle-aged; equally, 40–59 year olds (especially as women in their 40’s have markedly limited reproductive potential, they resemble more women in their 50’s who are post menopause, rather than women in their 20’s). Also, with aging. Older adults > 60 years realize weight redistribution, greater fat mass per BMI, and therefore are more alike than those in their 40’s. Educational attainments were classified as high school or below and college or above (some college, college graduate or above). Poverty index ratio (PIR) was the ratio of the family’s total income to the family’s appropriate poverty threshold calculated by the family’s mean income and number of individuals in their household. It was used to measure poverty status. A participant was considered poor if PIR < 1.0, and non-poor if PIR ≥ 1.0. Marital status was combined into three categories: never married, married or living with partner, and/or widowed, divorced or separated. Length of stay in the US was defined as follows. Participants were first asked whether they were born in the US; if they answered ‘no’, the length of time living in the US was recorded in years. Thus length of stay in the US was classified, in terms of years in the US, as 1) born in the US, 2) less than 10 years in the US, 3) 10 to 19 years in the US, and 4) 20 years or over.

### Statistical analysis

Data analyses were guided by the NHANES analytic and reporting guidance document [22]. Sampling weights and techniques were considered to account for oversampling and survey nonresponse. Student’s t tests with survey application were used to examine the significance of differences in the means of continuous variables, and chi-square tests were used for the significance of the proportions of categorical variables across central obesity status.

The prevalence of central obesity was calculated as the weighted number of participants with central obesity divided by the weighted number of participants in the study population. The prevalence estimates in different subgroups were age-adjusted by the direct method of standardization to 2010 US Census Asian population except for age-related domains. SURVEYFREQ and SURVEYREG procedures that take into account the survey design were used to estimate the prevalence with 95% confidence intervals (CIs) for subgroups of age, sex, education, poverty status, marital status, and length of time in the US. Similar procedures were applied to analysis of prevalence by the combination of sex and other characteristics. Chi-square tests were conducted to examine the significance of differences in the prevalence between subgroups. The difference was considered to be significant if the *p* value was less than 0.05. All data analyses were performed using SAS (version 9.3; SAS Institute Inc., Cary, NC).

## Results

Average age of Asian adults in the study sample was 44.8 years, and 20.4% were adults aged 60 years or over (Table 1). 53.0% were women, 26.1% had a high school education or below, and 86.6% were poor. 15.3% of Asian adults were born in the US. Compared with adults without central obesity, adults with central obesity were older (48.4 vs 40.8 years, *p* < .0001), more likely to be women (59.1% vs 44.3%, *p* < 0.0001), and more likely to be married or living with partner (73.6% vs 63.6%, *p* = 0.0007) and/or to be windowed, divorced or separated (12.5% vs 6.6%, *p* = 0.0068). Obese adults were also more likely to have lived in the US for more than 20 years and less likely to be born in the US.

**Table 1:** Characteristics of Asian Adults by Central Obesity Status in the United States, 2011–2014 (*n* = 1288)

Characteristics	N	Means or proportions (95% CI)			
		All	Central Obesity	Non-Central Obesity	*p*-value
Age (means), years	1288	44.8 (43.2–46.5)	48.4 (46.8–50.0)	39.8 (37.9–41.7)	<0.0001
Age group, %	1288				
20–39	496	42.1 (37.1–47.1)	32.6 (27.7–37.4)	55.7 (49.9–61.5)	
40–59	484	37.5 (33.9–41.1)	42.0 (38.6–45.4)	31.2 (26.4–36.0)	
≥ 60	308	20.4 (17.0–23.8)	25.5 (21.5–29.5)	13.1 (9.7–16.5)	<0.0001
Sex, %	1288				
Men	636	47.0 (45.2–48.8)	40.9 (38.6–43.1)	55.7 (52.5–59.0)	
Women	652	53.0 (51.2–54.8)	59.1 (56.9–61.4)	44.3 (41.0–47.5)	<0.0001
Education, %	1288				
High School or Below	366	26.1 (20.7–31.6)	28.1 (21.3–35.0)	23.3 (18.1–28.5)	
College or Above	922	73.9 (68.4–79.3)	71.9 (65.0–78.7)	76.7 (71.5–81.9)	0.09
Poverty status, %	1164				
Poor	169	86.6 (83.1–90.1)	87.6 (83.9–91.3)	85.3 (80.9–89.6)	
Non-poor	995	13.4 (9.9–16.9)	12.4 (8.7–16.1)	14.7 (10.4–19.1)	0.26
Marital Status, %	1287				
Never married	253	20.5 (16.2–24.8)	14.0 (10.7–17.2)	29.8 (23.5–36.1)	
Married or living with partner	899	69.4 (65.0–73.8)	73.6 (69.2–77.9)	63.5 (57.3–69.8)	
Widowed, divorced or separated	135	10.1 (8.4–11.7)	12.5 (9.9–15.0)	6.6 (4.1–9.2)	0.0005
Length of time in the US, %	1282				
Less than 10 years	304	24.5 (19.0–29.9)	22.1 (16.4–27.7)	27.9 (20.1–35.7)	
10 years to 19 years	293	23.0 (20.1–25.8)	23.7 (20.0–27.4)	21.9 (18.4–25.4)	
20 years or over	496	37.2 (32.7–41.7)	41.9 (36.9–46.8)	30.6 (26.1–35.2)	
Born in US	189	15.3 (12.3–18.4)	12.3 (8.7–16.0)	19.6 (15.4–23.8)	0.003

*Abbreviation*: *CI* confidence interval, *N* sample size

*P*-value indicates the significance of differences in the means or the proportions of characteristics across central obesity groups

**Fig. 1:**
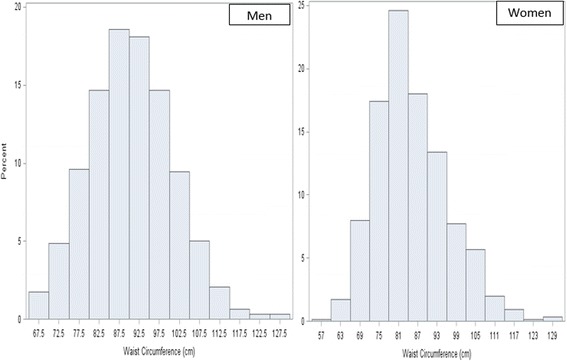
Histograms of waist circumference in Asian men and women in NHANES 2011–2014

The distributions of WC are presented in histogram separately for men and women, showing that WC had an approximate normal distribution in both men and women, and the average WC was higher in men than in women (Fig. 1). The average WC of Asian adults was 87.7 cm and did not change significantly over time during 2011 to 2014 (Table 2). The WC did not change over time across all groups of age, sex, education, marital status, and length of time in US. The overall prevalence of central obesity among US Asian adults was 58.1% in 2011–2014 (Table 3). The prevalence became higher with age from 45.4% in young adults to 73.5% in old adults (*p* < 0.0001). Women had 13.4% higher prevalence compared to men (64.4% vs 51.0%, *p* < 0.0001). The prevalence of central obesity increased in young people aged 20–39 years and in men. There was no significant difference over time in the prevalence of central obesity across the subgroups defined by marital status and length of stay in the US.

**Table 2:** Waist circumference mean over time among Asian Adults in the United States, 2011–2014 (*n* = 1288)

	Means (95% CI)			
	All	2011–2012	2013–2014	*p*-value
All	87.7 (86.8–88.6)	87.1 (85.8–88.5)	88.2 (87.2–89.2)	0.22
Age				
20–39 years	85.1 (83.8–86.3)	84.0 (82.0–86.1)	86.1 (84.7–87.4)	0.09
40–59 years	89.2 (88.3–90.2)	89.2 (87.8–90.5)	89.3 (87.9–90.7)	0.90
≥ 60 years	90.3 (88.9–91.7)	90.2 (89.1–91.4)	90.4 (87.9–92.9)	0.91
Sex				
Men	90.4 (89.5–91.3)	89.9 (88.6–91.4)	91.0 (89.8–92.2)	0.26
Women	85.0 (83.9–86.1)	85.5 (83.1–86.0)	85.5 (83.9–87.0)	0.47
Education				
High School or Below	87.4 (85.7–89.0)	88.2 (86.0–90.4)	86.5 (84.0–88.9)	0.37
College or Above	87.7 (86.8–88.5)	86.8 (85.8–87.8)	88.5 (87.4–89.6)	0.09
Poverty status				
Poor	86.4 (84.2–88.5)	87.0 (83.6–90.3)	85.8 (83.3–88.4)	0.47
Non-poor	87.8 (87.0–88.6)	87.1 (85.8–88.3)	88.5 (87.6–89.4)	0.11
Marital Status				
Never married	87.4 (85.6–89.3)	87.9 (85.4–90.4)	86.6 (83.8–89.3)	0.65
Married or living with partner	88.2 (87.2–89.1)	87.3 (86.1–88.5)	88.9 (87.6–90.2)	0.19
Widowed, divorced or separated	88.8 (85.1–92.5)	87.9 (84.7–91.0)	89.4 (83.9–95.0)	0.63
Length of time in the US				
Less than 10 years	87.6 (85.9–89.3)	86.7 (84.6–88.7)	88.8 (86.4–91.3)	0.20
10 to 19 years	87.5 (86.0–89.0)	88.0 (86.4–89.6)	87.1 (84.7–89.5)	0.49
20 years or over	88.6 (87.5–89.7)	87.9 (86.0–89.7)	89.4 (88.0–90.8)	0.23
Born in US	88.0 (86.0–90.1)	88.0 (85.5–90.6)	88.1 (85.1–91.0)	0.50

*Abbreviations*: *CI* confidence interval

Estimated means are age-adjusted by the direct standardization to the 2010 US census population except for age-related characteristics

*P*-value indicates the significance of differences in waist circumference means over time

**Table 3:** Prevalence and change of Central Obesity over time among Asian Adults in the United States, 2011–2014 (*n* = 1288)

	Prevalence (95% CI), %			
	All	2011–2012	2013–2014	*p*-value
All	58.1 (54.6–61.7)	55.3 (50.2–60.4)	60.9 (56.4–65.5)	0.54
Age				
20–39 years	45.4 (39.8–50.9)	39.2 (30.9–47.6)	51.5 (44.4–58.6)	0.0192
40–59 years	65.7 (62.6–68.7)	66.3 (62.6–70.0)	65.0 (59.9–70.2)	0.67
≥ 60 years	73.5 (67.8–79.1)	71.6 (65.0–78.3)	75.0 (65.7–84.4)	0.54
Sex				
Men	51.0 (47.1–54.8)	45.4 (40.0–50.8)	56.6 (52.3–60.9)	0.0095
Women	64.4 (60.2–68.6)	64.2 (57.8–70.6)	64.6 (59.0–70.1)	0.97
Education				
High School or Below	58.0 (50.7–65.2)	56.1 (48.6–63.7)	58.2 (46.1–70.3)	0.90
College or Above	58.0 (54.3–61.7)	54.2 (49.0–59.3)	61.7 (57.0–66.3)	0.0476
Poverty status				
Poor	54.1 (46.4–61.7)	55.3 (45.9–64.8)	52.1 (40.6–63.7)	0.61
Non-poor	59.2 (55.7–62.6)	55.4 (50.0–60.8)	62.4 (58.2–66.7)	0.0468
Marital Status				
Never married	57.9 (49.7–66.1)	59.3 (49.7–68.9)	55.0 (43.6–66.3)	0.59
Married or living with partner	61.0 (56.5–65.4)	57.7 (51.3–64.1)	63.6 (57.8–69.5)	0.25
Widowed, divorced or separated	63.2 (47.7–78.7)	64.1 (43.6–84.6)	63.1 (42.1–84.1)	0.99
Length of time in the US				
Less than 10 years	58.6 (50.3–66.8)	54.9 (45.7–64.1)	62.8 (49.6–76.0)	0.51
10 to 19 years	60.0 (55.3–64.7)	63.2 (56.4–70.1)	57.0 (50.5–63.5)	0.15
20 years or over	63.7 (58.8–68.6)	60.1 (50.8–69.3)	66.9 (62.3–71.5)	0.10
Born in US	52.7 (43.5–61.9)	51.7 (41.2–62.2)	53.8 (39.9–67.8)	0.76

*Abbreviations*: *CI* confidence interval

Estimated prevalence is age-adjusted by the direct standardization to the 2010 US census population except for age-related characteristics

*P*-value indicates the significance of differences in the prevalence of central obesity over time

The prevalence of central obesity was significantly higher in older adults in both men and women (*p* < 0.0001) (Table 4). Compared with men, women had higher prevalence in each subgroup of age, education, poverty, and length of time (except for the subgroup of “born in the US”) (all *p* < 0.05) and in the subgroup of “married or living with partner” for marital status (*p* < 0.0001). The prevalence was 8.5% higher in young women vs young men (*p* = 0.0256), 16.7% higher in middle-aged women vs middle-aged men (*p* < 0.0001), and 18.5% higher in old women vs old men (*p* < 0.0001). Women with college education or above had 12.4% higher, and women with high school education or below had 17.2% higher prevalence of central obesity than the corresponding men. The prevalence was 11.9% higher in non-poor women vs non-poor men (*p* = 0.0002), and 30.5% higher in poor women vs poor men (*p* < 0.0001). Women with length of stay in the US less than 10 years, 10–19 years, and more than 20 years had 10.6%, 13.3%, and 21.8% higher prevalence, respectively, than men in the same group.

**Table 4:** Prevalence of Central Obesity by sex among Asian Adults in the United States, 2011–2014 (*n* = 1288)

Characteristics	Prevalence (95% CI), %		
	Men	Women	*p*-value
Age			
20–39 years	41.0 (34.7–47.3)	49.5 (42.5–56.5)	0.0256
40–59 years	56.9 (52.2–61.6)	73.6 (68.9–78.3)	<0.0001
≥ 60 years	62.8 (56.3–69.3)	81.3 (73.7–88.9)	<0.0001
Education			
High School or Below	49.0 (41.3–56.6)	66.2 (55.4–76.9)	0.0005
College or Above	51.5 (46.8–56.2)	63.9 (59.3–68.6)	<0.0001
Poverty status			0.0108
Poor	37.1 (28.5–45.7)	67.6 (55.5–79.7)	<0.0001
Non-poor	52.7 (48.3–57.1)	64.6 (60.2–69.1)	0.0002
Marital Status			
Never married	57.4 (43.2–71.6)	57.6 (48.4–66.9)	0.99
Married or living with partner	52.9 (46.9–58.9)	68.2 (63.1–73.2)	<0.0001
Widowed, divorced or separated	54.8 (33.6–76.1)	66.1 (48.7–83.5)	0.17
Length of time in the US			
Less than 10 years	52.8 (43.3–62.3)	63.4 (53.4–73.3)	0.0483
10 to 19 years	52.1 (45.1–59.2)	65.4 (60.0–70.8)	0.0012
20 years or over	52.2 (44.1–60.4)	74.0 (66.1–81.9)	0.0002
Born in US	46.4 (32.9–60.0)	58.2 (45.7–70.6)	0.08

*Abbreviations*: *CI* confidence interval

Estimated prevalence is age-adjusted by the direct standardization to the 2010 US census population except for age-related characteristics

*P*-value indicates the significance of differences in the prevalence of central obesity between men and women across other characteristics groups

## Discussion

We used the IDF criteria for Asian populations to define central obesity by WC among US Asian adults. Our analysis showed that the prevalence of central obesity was 58.1% among Asian adults. The result is not consistent with the prevalence estimate in Asian adults from a previous report that utilized the same data [18]. The cause of inconsistency is that the investigators in the previous study used the guidelines from the Adult Treatment Panel (ATP) III of the National Cholesterol Education Program to define central obesity. ATP III adopts the cut-off points of WC ≥ 88 cm for women and ≥102 cm for men for the diagnosis of central obesity [23]. These criteria are used for all US racial/ethnic groups for clinical diagnosis and epidemiological studies [16, 18]. The criteria from ATP III underestimates the prevalence of central obesity among Asian individuals. IDF recommends that ethnic group specific cut-points be applied when defining central obesity for individuals from different racial/ethnic groups. The IDF criteria for the diagnosis of central obesity has been shown to be more useful in identifying Asian individuals with higher risk of metabolic syndrome [24–26].

Although the prevalence of general obesity calculated from BMI is much lower among Asians than other racial/ethnic groups in the US [23], the prevalence of central obesity characterized by WC is similar across racial/ethnic groups (58.1% in Asians from our study, and 53.8% in non-Hispanic Whites, 57.4% in Mexican Americans, and 60.9% in non-Hispanic Blacks from the previous report [18]). Despite lower BMIs, Asians have greater amounts of visceral fat for every level of BMI compared to non-Hispanic whites [27, 28]. The morbidities related to excess adiposity occur more frequently at lower BMI levels in Asians than in whites [29, 30]. This phenomenon is partially explained by excess body fat, specifically higher intra-abdominal and subcutaneous fat, and ectopic fat deposition which are associated with higher risk of dyslipidemia, diabetes, and hypertension in Asians [29].

The prevalence of central obesity was higher while the average WC was lower in Asian women than in men. The paradox is mostly due to the different cutoffs we used for men and women when defining central obesity by WC. The higher prevalence in Asian women coincides with the findings in the populations of whites, blacks, and Mexican Americans. [18] Our analysis further showed that women had higher prevalence than men in each subgroup of age, education, poverty, and length of stay in the US. Similar results were also found in other ethnic populations [16, 18, 31]. The cut-off values of WC for diagnosis of central obesity in men and women vary (80 cm for women vs 90 cm for men). Men have close to twice the visceral fat as that of pre-menopausal women, and women have higher subcutaneous fat accumulation compared with men [4, 5]. The higher rate of central obesity in Asian women may be attributed to excess subcutaneous fat rather than visceral fat. Due to higher prevalence among Asian men (compared to men in other racial/ethnic groups), the sex difference in central obesity rate is smaller in Asians than in other racial/ethnic groups (Asians, 13.4%; non-Hispanic whites, 18.8%; non-Hispanic blacks, 35.4%; Mexican Americans, 28.4%) [20]. Several Asian studies indicates higher prevalence of metabolic syndrome among Asian men than women, although women have higher central obesity rate than men [24, 32]. Considering the rise in central obesity rate among Asian men, we predict that the risk of morbidities related to central obesity will increase in the male population.

The overall prevalence of central obesity among US Asian adults did not change over time, whereas the prevalence in whites, blacks, and Mexican Americans is increasing [18]. The prevalence of central obesity among Asians did increase over time in young adults, men, and adults with a college education or above. The studies using the same NHANES data showed that large increases of central obesity rate were found in young to middle-aged men, and Africa American men in the last decade [16, 31]. However, the relative increase of young Asian adults and Asian men in this study were much higher than other ethnic groups (young Asian adults: 31.4%; Asian men: 24.7%; non-Hispanic Black men (2009–2012): 5.1%) [18]. Although the central obesity rate for Asian women did not increase significantly, women have higher rates than men in each subgroup.

The prevalence of central obesity was higher in non-poor men than in poor men, while the prevalence was lower in non-poor than in poor women. The educational attainment and poverty status parallel socioeconomic status. Although the reason for differential correlations between socioeconomic status and the prevalence of central obesity by sex is not clear, our results are consistent with the findings from other obesity studies [16, 33]. However, the expected relationships between socioeconomic status and obesity seem to be attenuated in recent NHANES surveys [16].

The prevalence of obesity and central obesity differed over marital status. Several studies have indicated that married adults had higher rate of overweight or obesity than other marital status groups combined, and never-married people had lower obesity rate than married people [34, 35]. Other investigators have shown that transitions into marriage were associated with weight gain, whereas transitions out of marriage were associated with weight loss [36]. Our results indicate that married women had higher prevalence of central obesity than non-married women. The prevalence of central obesity became higher with years of stay in the US among first-generation Asian women. However, Asian women who were born in the US has the lowest prevalence of central obesity. It is likely that the increased energy intake of western diet: fast and convenience food, sugar sweetened beverage, and high fat/high sugar food has contributed to the prevalence of obesity or central obesity among Asian immigrants [37]. High socioeconomic status and less manual work have also contributed to greater sedentariness among Asian immigrants [8]. In addition, immigration itself may result in stress and increased consumption of alcohol and food [28]. Native-born immigrants may be more acculturated and tend to be more physically active than first generation [37]. Dietary and physical activities of western lifestyle may be associated with the prevalence of central obesity among American Asians in different directions.

NHANES is a national survey designed to monitor the health and nutrition status among adults and children in the US. The results in this study are good representation of the prevalence of central obesity among US Asian adults at the national level. The prevalence estimates were age-adjusted by the method of direct standardization to the 2010 US census population. The age-adjusted method reduces the bias in crude rates that results from the difference of age distributions across groups. Age-adjusted prevalence is more accurate to reflect the real prevalence due to controlling for the confounding impact of age.

There are a few limitations in the study. NHANES started collecting data of US Asians in 2011. The relatively small sample size was a concern, and might bias the prevalence estimates for some subgroups of characteristics (e.g. marital status and length of time in US) with limited statistical power to test the significance of differences in the prevalence of central obesity. Data of Asians were only available in two phases of 2011–2012 and 2013–2014. Linear trends in the prevalence of central obesity could not be evaluated whereas the differences were examined in the prevalence between 2011 and 2012 and 2013–2014. NHANES surveys did not collect data of country of origin in Asian immigrants. The lack of geographic information for diverse Asian populations, such as Chinese, Japanese, Indian, and Vietnamese, likely masked the prevalence difference in these distinct ethnic Asian groups. In the future, we would aspire to assist the NHANES program sponsored by NCHS in CDC to disaggregate Asian American groups to address this limitation for future studies.

## Conclusions

This study highlighted the public health problem of central obesity in US Asians, a rapidly growing segment of the US population. Our study revealed a relatively high prevalence of central obesity among US Asian adults, despite the lower prevalence of general obesity (based on BMI) as compared to other racial/ethnic groups reported from previous studies. Monitoring central obesity may provide additional information for accurately predicting all-cause and obesity-related mortality and morbidity, and support interventions in clinical practice and public health campaigns, to address these disparities and target the Asian population who have been largely ignored by virtue of using a single metric (i.e. BMI). Efforts are needed to promote applying the measurement of WC in primary care practice to classify at-risk US Asian adults in order to reduce the risks of adverse outcomes. Our results support the routine measurement of WC in clinical care for Asian adults, consistent with current recommendations as a key step in initiating the prevention, control, and management of central obesity among US Asians.

## Abbreviations

ATP: Adult Treatment Panel
BMI: body mass index
CDC: Centers for Disease Control and Prevention
CI: confidence interval
CVD: cardiovascular disease
IDF: International Diabetes Federation
NCHS: National Center for Health Statistics
NHANES: National Health and Nutrition Examination Survey
PIR: poverty index ratio
US: United States
WC: waist circumference

## References

[1] Kochanek KD, Xu JQ, Murphy SL, Miniño AM, Kung HC (2011). Deaths: final data for 2009. National vital statistics reports. Vol 61 no 4.

[2] Zaninotto P, Pierce M, Breeze E, de Oliveira C, Kumari M (2010). BMI and waist circumference as predictors of well-being in older adults: findings from the English longitudinal study of ageing. Obesity.

[3] Rankinen T, Kim SY, Pérusse L, Despres JP, Bouchard C (1999). The prediction of abdominal visceral fat level from body composition and anthropometry: ROC analysis. Int J Obes Relat Metab Disord.

[4] Pouliot MC, Després JP, Lemieux S, Moorjani S, Bouchard C, Tremblay A (1994). Waist circumference and abdominal sagittal diameter: best simple anthropometric indexes of abdominal visceral adipose tissue accumulation and related cardiovascular risk in men and women. Am J Cardiol.

[5] Koster A, Leitzmann MF, Schatzkin A, Mouw T, Adams KF, JTM v E (2008). Waist circumference and mortality. Am J Epidemiol.

[6] Jacobs EJ, Newton CC, Wang Y, Patel AV, McCullough ML, Campbell PT (2010). Waist circumference and all-cause mortality in a large US cohort. Arch Intern Med.

[7] Clinical Guidelines on the Identification, Evaluation, and Treatment of Overweight and Obesity in Adults--The Evidence Report. National Institutes of Health. Obes Res. 1998;6(Suppl 2):51S–209S. 9813653

[8] Mohan V, Deepa R, Rani SS, Premalatha G (2001). Prevalence of coronary artery disease and its relationship to lipids in a selected population in South India: the Chennai urban population study (CUPS no. 5). J Am Coll Cardiol.

[9] Zaman MM, Yoshiike N, Rouf MA (2001). Cardiovascular risk factors: distribution and prevalence in a rural population of Bangladesh. J Cardiovasc Risk.

[10] Kanaya AM, Kandula N, Herrington D, Budoff MJ, Hulley S, Vittinghoff E (2013). Mediators of atherosclerosis in south Asians living in America (MASALA) study: objectives, methods, and cohort description. Clin Cardiol.

[11] Hsu WC, Araneta MRG, Kanaya AM, Chiang JL, Fujimoto W (2015). BMI cut points to identify at-risk Asian Americans for type 2 diabetes screening. Diabetes Care.

[12] Rajpathak SN, Wylie-Rosett J (2011). High prevalence of diabetes and impaired fasting glucose among Chinese immigrants in new York City. J Immigr Minor Health.

[13] Bernstein R. Asians Fastest-Growing Race or Ethnic Group in 2012, Census Bureau Reports. For Immediate Release. US Census Bureau. Available at: http://www.census.gov/newsroom/press-releases/2013/cb13-112.html. Accessed 15 Jan 2016.

[14] Ladabaum U, Mannalithara A, Myer PA, Singh G (2014). Obesity, abdominal obesity, physical activity, and caloric intake in US adults: 1988-2010. Am J Med.

[15] Gutiérrez-Fisac JL, Guallar-Castillón P, León-Muñoz LM, Graciani A, Banegas JR, Rodríguez-Artalejo F (2012). Prevalence of general and abdominal obesity in the adult population of Spain, 2008-2010: the ENRICA study. Obes Rev.

[16] Li C, Ford ES, McGuire LC, Mokdad AH (2007). Increasing trends in waist circumference and abdominal obesity among US adults. Obesity (Silver Spring).

[17] Xi B, Liang Y, He T, Reilly KH, Hu Y, Wang Q (2012). Secular trends in the prevalence of general and abdominal obesity among Chinese adults, 1993-2009. Obes Rev.

[18] Ford ES, Maynard LM, Li C (2014). Trends in mean waist circumference and abdominal obesity among US adults, 1999-2012. JAMA.

[19] Continuous NHANES Web Tutorials: Sampling Design. Centers for Disease Control and Prevention. Available at: https://www.cdc.gov/nchs/tutorials/nhanes/surveydesign/SampleDesign/intro.htm. Accessed 3 July 2017.

[20] NHANES: Anthropometry Procedures Manual. Centers for Disease Control and Prevention. Available at: http://www.cdc.gov/nchs/data/nhanes/nhanes_11_12/Anthropometry_Procedures_Manual.pdf. Accessed 2 Oct 2016.

[21] The IDF Consensus Worldwide Definition of the Metabolic Syndrome. International Diabetes Federation. Available at: http://www.idf.org/webdata/docs/MetSyndrome_FINAL.pdf. Accessed 2 Oct 2016.

[22] National Health and Nutrition Examination Survey: Analytic Guidelines, 2011–2012. Centers for Disease Control and Prevention. Available at: http://www.cdc.gov/nchs/nhanes/survey_methods.htm. Accessed 10 Oct 2016.

[23] National Cholesterol Education Program (NCEP) Expert Panel on Detection, Evaluation, and Treatment of High Blood Cholesterol in Adults (Adult Treatment Panel III) (2002). Third report of the National Cholesterol Education Program (NCEP) expert panel on detection, evaluation, and reatment of high blood cholesterol in adults (adult treatment panel III) final report. Circulation.

[24] Tan CE, Ma S, Wai D, Chew SK, Tai ES (2004). Can we apply the National Cholesterol Education Program Adult Treatment Panel definition of the metabolic syndrome to Asians?. Diabetes Care.

[25] Mirrakhimov AE, Lunegova OS, Kerimkulova AS, Moldokeeva CB, Nabiev MP, Mirrakhimov EM (2012). Cut off values for abdominal obesity as a criterion of metabolic syndrome in an ethnic Kyrgyz population (central Asian region). Cardiovasc Diabetol.

[26] Ogden CL, Carroll MD, Kit BK, Flegal KM (2014). Prevalence of childhood and adult obesity in the United States, 2011-2012. JAMA.

[27] Banerji MA, Faridi N, Atluri R, Chaiken RL, Lebovitz HE (1999). Body composition, visceral fat, leptin, and insulin resistance in Asian Indian men. J Clin Endocrinol Metab.

[28] Misra A, Bhardwaj S (2014). Obesity and the metabolic syndrome in developing countries: focus on south Asians. Nestle Nutr Inst Workshop Ser.

[29] Misra A, Khurana L (2008). Obesity and the metabolic syndrome in developing countries. J Clin Endocrinol Metab.

[30] Vikram NK, Pandey RM, Misra A, Sharma R, Devi JR, Khanna N (2003). Non-obese (body mass index < 25 kg/m2) Asian Indians with normal waist circumference have high cardiovascular risk. Nutrition.

[31] Ford ES, Li C, Zhao G, Tsai J (2011). Trends in obesity and abdominal obesity among adults in the United States from 1999-2008. Int J Obes (Lond).

[32] Khan SA, Jackson RT (2015). The prevalence of metabolic syndrome among low-income south Asian Americans. Public Health Nutr.

[33] Qin X, Zhang Y, Cai Y, He M, Sun L, Fu J (2013). Prevalence of obesity, abdominal obesity and associated factors in hypertensive adults aged 45-75 years. Clin Nutr.

[34] Schoenborn CA (2004). Marital status and health: United States, 1999–2002. Adv Data.

[35] Memish ZA, El Bcheraoui C, Tuffaha M, Robinson M, Daoud F, Jaber S (2014). Obesity and associated factors — Kingdom of Saudi Arabia, 2013. Prev Chronic Dis.

[36] Dinour L, Leung MM, Tripicchio G, Khan S, Yeh MC (2012). The association between marital transitions, body mass index, and weight: a review of the literature. J Obes.

[37] Albrecht SS, Gordon-Larsen P (2013). Ethnic differences in body mass index trajectories from adolescence to adulthood: a focus on Hispanic and Asian subgroups in the United States. PLoS One.

